# A Closed-Loop Audit: The Assessment of Red Flags and Management of Acute Conjunctivitis in Primary Care

**DOI:** 10.7759/cureus.83735

**Published:** 2025-05-08

**Authors:** Monica Kelada

**Affiliations:** 1 Primary Care, Dapdune House Surgery, Guildford, GBR

**Keywords:** acute red eye, assessment for sight-threatening conditions, conjunctivitis, ophthalmology, primary care

## Abstract

Background

Acute red eye is a common presenting complaint to primary care. Although conjunctivitis is the most common cause, some conditions can be sight-threatening. Diagnosing such conditions can be difficult given the broad differential diagnoses and limited specialist equipment. The National Institute for Health and Care Excellence (NICE) has published five "red flags," which may indicate the need for urgent ophthalmological assessment: reduced visual acuity (VA), copious discharge, marked eye pain/photophobia, contact lens use and recent trauma. For patients not requiring specialist referral, NICE recommends conservative management and reserving antibiotics for non-resolving/severe cases.

Aims

The primary objective of this audit was to improve the assessment and documentation of acute red eye in primary care. The secondary outcome was to evaluate management appropriateness.

Methodology

We conducted a closed-loop audit to evaluate the clinical assessment and management of acute red eye within a single primary care practice. All cases coded as "conjunctivitis" or "acute red eye" over a one-year period were included. The following aspects of the clinical assessment were explored: symptom duration, laterality and red flag assessment. The conjunctivitis type diagnosed (bacterial, viral, allergic or unspecified) and management strategies were recorded.

The audit introduced a multi-faceted intervention to improve the assessment and management of red flag symptoms in patients presenting with acute red eye. A practice meeting was conducted to raise awareness about the importance of assessing for red flag symptoms and adhering to guidelines. Additionally, a standardised template for GPs to use during consultations and a text message patient questionnaire for telephone consultations were implemented. The impact of these implementations was reassessed after one year.

Results

Over a one-year period, 42 cases were identified. On average, patients presented after 2.4 days of symptoms. Overall, 97.6% (41/42) documented symptom laterality. On average, each patient had 1.8 red flags assessed. One patient had red flag symptoms and was correctly referred to same-day ophthalmology services. Out of 42 patients, 41 were diagnosed with unspecified conjunctivitis, while one was diagnosed with viral conjunctivitis. Although there were zero recorded cases of bacterial aetiology, 73.8% (31/42) were prescribed antibiotics and 16.7% (7/42) were given hygiene advice and a deferred antibiotic drops script, while only 7.1% (3/42) were managed conservatively.

Post-interventions, 13 cases were identified. On average, each patient had 3.8 red flags assessed (p<0.001). Only one patient was identified as having red flag symptoms and reduced VA and was referred to ophthalmology services. Out of the 13 patients, 12 were diagnosed with unspecified conjunctivitis, while one was diagnosed with bacterial aetiology; 15.3% (2/13) were managed conservatively, while the remaining 84.6% (11/13) were prescribed antibiotics. Two out of the 11 patients given antibiotics were prescribed fusidic acid, having been refractory to chloramphenicol.

Conclusion

This audit demonstrated that significant improvements in clinical assessment of conjunctivitis can be achieved through practical and inexpensive interventions. However, antibiotic prescribing remained high despite limited bacterial diagnoses. Further efforts are needed to sustain improvements and reduce unnecessary antibiotic use.

## Introduction

Acute red eye is a common presenting complaint to primary care physicians [1]. Red eye is the cardinal sign of ocular inflammation [2]. In most cases, the condition is usually benign and can be managed in primary care. Conjunctivitis, the most common cause of red eye, may have a non-infectious (e.g., allergies and irritants) or infectious (e.g., viral and bacterial) aetiology.

Although less common, causes of red eye, such as iritis, keratitis and acute angle-closure glaucoma, can pose a serious threat to vision if not promptly diagnosed and treated [3]. Studies have indicated that up to 64% of patients presenting with red eye in primary care are incorrectly diagnosed, with 10% of these cases resulting in serious clinical consequences [4]. Recognising such conditions can be difficult given the broad differential diagnoses and limited specialist equipment [1]. The National Institute for Health and Care Excellence (NICE) recommends that all patients be assessed for potentially sight-threatening signs to aid the diagnosis of such conditions. NICE has published five "red flags," which may indicate the need for urgent ophthalmic assessment. These include marked eye pain/photophobia, reduced visual acuity (VA), contact lens use, copious discharge and recent trauma [5].

The five red flag symptoms highlighted by NICE can have serious clinical implications if present. Marked pain or photophobia may indicate uveitis or keratitis, both of which can lead to vision loss if untreated [6]. Reduced visual acuity can suggest conditions such as acute angle-closure glaucoma, orbital cellulitis and herpes simplex virus keratitis, all of which require urgent intervention to prevent permanent vision loss [7]. Through inappropriate use or care, contact lens wearers are at increased risk for *Acanthamoeba* keratitis, a rapidly progressive infection that can perforate the cornea [7,8]. Recent ocular trauma can introduce pathogens or cause structural damage, predisposing to secondary infections or globe rupture. Copious purulent discharge may be a sign of hyperacute bacterial conjunctivitis, often caused by *Neisseria gonorrhoeae*, which can rapidly damage the cornea [5].

For patients who do not require specialist referral, NICE recommends avoiding antibiotics. Several trials have confirmed a high rate of disease resolution without antibiotics [9,10]. With a lack of clinical efficacy in the majority of cases, antibiotics also come with the risk of adverse side effects and bacterial resistance when used inappropriately. In 2024, the Royal College of Ophthalmologists, in collaboration with the UK Ophthalmic Pharmacy Group, declared a shortage of chloramphenicol in the United Kingdom [11]. This further necessitates the need for careful consideration before antibiotic prescription or recommendation.

The primary objective of this audit was to evaluate the assessment for and documentation of red flag features for patients presenting with conjunctivitis in primary care and compare this to the standard that all patients should be assessed for these features. The secondary outcome was to evaluate the appropriateness of management. With the aim of improving the assessment, documentation and management of conjunctivitis, a multi-faceted intervention was launched, and the first cycle of this audit was closed after one year.

## Materials and methods

This closed-loop audit was conducted in a single primary care centre in the United Kingdom using their electronic patient record system, Egton Medical Information Systems (EMIS) (EMIS Health, Leeds, United Kingdom). Within EMIS, all patient consultations coded as "conjunctivitis" or "acute red eye" over a one-year period (between October 2022 and 2023) were included. Patients under the age of 18 were excluded. All patient details were removed and anonymised.

The following documentation was extracted manually directly from documented consultation notes in EMIS and organised using Microsoft Excel (Microsoft Corporation, Redmond, WA): laterality and duration of symptoms, assessment of the five red flag features and management. If a red flag feature had been assessed, it was noted if it was a positive/negative finding and if a same-day ophthalmology referral was made where appropriate.

A multi-faceted intervention was implemented. Interventions included a practice meeting with general practitioners (GPs), physician associates and pharmacists to raise awareness about the importance of assessing for red flag symptoms and adhering to clinical guidelines. Additionally, a standardised template for healthcare professionals to use during consultations was launched on EMIS (Appendices). For remote consultations, a text message questionnaire for patients to fill in prior to the consultation was launched on Accurx (Accurx, London, United Kingdom) (Appendices). Both the EMIS template and Accurx text were developed using the NICE red flag symptoms and set up by the practice IT manager.

The impact of these interventions (clinical assessment and management) was assessed after one year. Patients seen between January 2024 and August 2024 were included in the re-audit.

Data analysis was undertaken using the online calculator GraphPad (GraphPad Software, La Jolla, CA). Non-parametric data was analysed using the Mann-Whitney U test and a Z-test.

## Results

Over a one-year period, 42 cases were identified (Table 1). The most commonly asked question was the lateralisation of their symptoms, asked 97.6% of the time at baseline. On average, patients presented after 2.4 days of symptoms.

**Table 1:** Baseline demographics of the study population Mann-Whitney test: age, Z-test: sex ns: non-significant, SD: standard deviation

	Pre-intervention	Post-intervention	p-value
Age (mean (±SD)) (year)	52±23.6	39±10.1	ns
Sex			
Male (number (%))	15 (35.7)	3 (23.1)	ns
Female (number (%))	27 (64.3)	10 (76.9)	ns

On average, 1.8 red flags were assessed per patient. The most frequently documented red flag was the evaluation of discharge quantity, recorded in 88.1% of consultations. Marked pain or photophobia was assessed in 47.6% of cases, followed by reduced VA in 33.3%, contact lens use in 7.14% and history of trauma in 2.38% of consultations.

Only one patient had a red flag symptoms identified. The patient had two positive red flags (marked eye pain and recent contact lens use). This patient was correctly referred to same-day ophthalmology services and was found to have bilateral microbial keratitis.

Upon review of diagnosis and management, 41 out of 42 patients were diagnosed with unspecified conjunctivitis, while one was diagnosed with viral conjunctivitis. Although there were zero recorded cases of bacterial aetiology, 73.8% of cases were prescribed antibiotics and 16.7% were given hygiene advice and a deferred script for antibiotic drops. Although being the first line recommended management by NICE, only 7.1% were managed conservatively.

**Figure 1:**
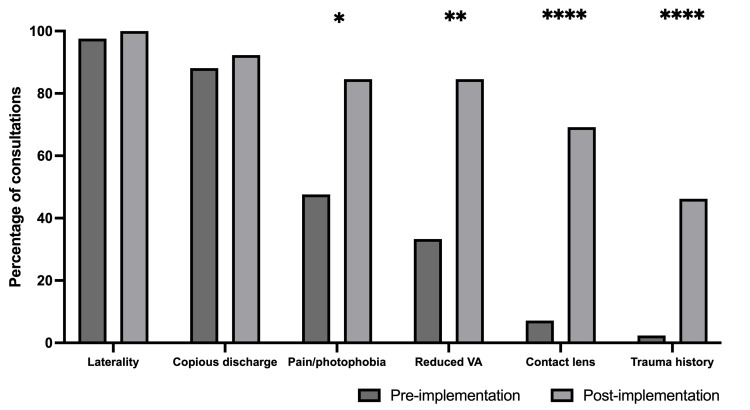
Percentage of consultations assessing each red flag feature Dark grey: pre-audit implementations (n=42), light grey: post-audit implementations (n=13) VA: visual acuity Z-test: *p≤0.05, **p≤0.01, ****p≤0.0001

Following the implementation of the interventions, 13 patients were included in the study (Table 1), with six cases receiving face-to-face reviews and seven receiving remote consultations. There was a significant improvement in the average number of red flag symptoms assessed and documented. On average, 3.88 red flags were assessed per patient (p<0.001). The most frequently documented red flag was the evaluation of discharge quantity, recorded in 92.3% of consultations. Marked pain or photophobia and reduced visual acuity were both assessed in 84.6% of cases, followed by contact lens use in 69.2% and history of trauma in 46.2% of consultations (Figure 1).

During this post-intervention period, only one (out of the 13 patients) was identified as having red flag symptoms, specifically reduced VA. This patient was appropriately referred to ophthalmology services for same-day specialist evaluation. Their final diagnosis, however, was not sight-threatening, and they were diagnosed with bacterial conjunctivitis. Their reduced VA was attributed to their experience of discharge.

Out of the 13 patients reviewed during the post-intervention phase, 12 were diagnosed with unspecified conjunctivitis, while one case was attributed to bacterial aetiology. Despite the low rate of confirmed bacterial conjunctivitis, antibiotic prescribing remained high. A total of 84.6% of patients (11 out of 13) were prescribed antibiotics, while 15.3% were managed conservatively without antibiotic treatment. Amongst those who received antibiotic therapy, two patients were prescribed fusidic acid after failing to respond to initial treatment with chloramphenicol.

## Discussion

Acute red eye is a common presentation in primary care. While conjunctivitis is the most common underlying cause, it is important for clinicians to be able to identify serious eye conditions that warrant referral. This closed-loop audit demonstrated that practical and low-cost interventions, such as clinician education, consultation templates and patient questionnaires, can significantly improve the assessment and documentation of red flag symptoms in patients presenting with acute red eye in primary care. Our findings suggest, however, a continued preference for medical management over watchful waiting or conservative treatment.

The most notable improvement was the increased documentation of the five NICE red flag features. Our findings corroborate previous studies where structured documentation tools significantly increased the identification of high-risk clinical features [12]. Hayre's study demonstrated a significant improvement in red flag documentation following a doctor training session and the implementation of an EMIS template [12]. Similarly, Kilduff and Lois also reported a significant improvement in the average number of red flag features assessed after a primary care teaching session (0.9 pre-intervention versus 2.7 post-intervention, p<0.001) [13].

Toolkits have also been shown to be greatly beneficial in patient assessment [13,14]. Kilduff and Lois implemented a "red eye survival toolkit" in each consultation room that contained a Snellen chart, a guide to the Snellen chart, a tape measure and a guide to eye-drop application. Authors suggested that permanent reminders of the red flag features and having an assessment modality for visual acuity can help improve patient assessment [13].

Across both the pre- and post-implementation periods, one patient in each group was identified with red flag symptoms and appropriately referred to same-day ophthalmology services. Pre-implementation, the patient presented with marked eye pain and recent contact lens use and was diagnosed with bacterial keratitis. Post-implementation, the identified red flag was copious ocular discharge, potentially indicative of a serious infection such as *Neisseria gonorrhoeae*. After specialist assessment, this was ruled out, and the patient was discharged with a follow-up arranged via their GP.

Although the overall number of red flag findings remained the same, the structured checklist introduced post-implementation likely played a significant role in the second case. Copious discharge can be a subjective symptom and may not have been actively asked about or documented prior to the intervention. This highlights the importance of standardised red flag assessments, not just for increasing detection but for ensuring consistency in history-taking, reducing variation in clinical judgement and safeguarding against missed or delayed recognition of potentially serious pathology.

The Royal College of General Practitioners (RCGP) states that the role of the GP is to "take a focused history, examine, diagnose and treat common eye conditions and know when to refer to specialist care" [15]. Studies, however, have demonstrated a lack of confidence surrounding ophthalmic cases among GPs, with Featherstone et al. reporting 57% of GPs not feeling confident and 80% requesting informal teaching [16]. In the United Kingdom, a clinical placement in ophthalmology is not a mandatory requirement of the medical curriculum, and only 6% of GPs have worked in ophthalmology [17,18]. Hence, the majority rely on undergraduate knowledge, which is suboptimal for postgraduate purposes. It is, therefore, arguable that inadequate assessment and poor management in primary care stem from undergraduate training that may benefit from reform.

Assessing red flag symptoms may have helped GPs feel more confident in managing patients with negative findings, which, in turn, could theoretically have led to a reduction in antibiotic use. Despite improved red flag assessment post-implementation, antibiotic prescribing remained high. This continued overprescription contrasts with NICE guidelines, which recommend conservative management for most cases of conjunctivitis, given the high rate of spontaneous resolution and that viral conjunctivitis is the most common cause of infectious conjunctivitis in adults [5,10,19]. Overprescribing is not only clinically unnecessary but also contributes to antimicrobial resistance, induces side effects and increases costs [19]. This issue is particularly concerning given the recent chloramphenicol shortage declared by the Royal College of Ophthalmologists in 2024 [11]. The limited availability of first-line treatments emphasises the need for more judicious prescribing practices.

Although NICE guidelines advocate for conservative management in the absence of red flag symptoms, this approach was underutilised. Prior to the intervention, only 7.1% of patients were managed conservatively, increasing modestly to 15.3% post-intervention. Deferred prescriptions, a strategy known to reduce unnecessary antibiotic use while providing treatment if symptoms worsen, were issued to only 16.7% of patients in the initial phase and were not reported post-intervention. The absence of deferred prescribing post-intervention may reflect a change in clinician behaviour following the implementation of the new guidance, which emphasised the self-limiting nature of most conjunctivitis cases and encouraged non-antibiotic management. It is possible that clinicians, having gained greater confidence in withholding antibiotics altogether, opted not to offer deferred prescriptions as a middle ground. However, we cannot determine definitively whether this shift was intentional or simply not consistently documented, and we have now acknowledged this limitation in the discussion. Our findings surrounding conjunctivitis management suggest an opportunity to further educate clinicians on alternative management strategies.

Despite national guidance recommending a conservative approach to antibiotic use in conjunctivitis, overprescribing remains common in primary care. Several factors may contribute to this. Diagnostic uncertainty can lead clinicians to prescribe antibiotics as a precaution, particularly in the absence of access to slit-lamp examination or microbiological testing [16]. Time pressures during short consultations may discourage in-depth explanations about the self-limiting nature of most cases, leading clinicians to offer antibiotics to expedite care. Additionally, perceived or real patient expectations may influence prescribing behaviour, with some patients expecting a prescription to validate their consultation [20]. These factors highlight the complexity of prescribing decisions and underscore the need for continued education, support and system-level interventions to promote antimicrobial stewardship.

While this audit demonstrated immediate improvements in assessment and documentation, sustaining these changes over time remains a challenge. Studies have shown that without ongoing reinforcement, clinician adherence to guidelines often declines [21]. Future quality improvement initiatives could include periodic training sessions, assessment toolkits, regular audit cycles and continuous integration of decision support tools to maintain high standards of care.

A key strength of this audit is its closed-loop design, allowing the assessment of both pre- and post-intervention outcomes. However, there are several limitations. The audit was conducted in a single primary care practice, limiting the generalisability of findings. The sample size was relatively small, particularly post-intervention (n=13), reducing statistical power. Additionally, the audit relied on clinician documentation, which may not fully capture all assessments performed. It was not possible to definitively determine whether the EMIS template or Accurx questionnaire was used in each post-intervention consultation, although the structure of documentation often suggested their use. The mode of consultation was recorded, with the assumption that face-to-face reviews were more likely to use the EMIS template and telephone reviews the Accurx questionnaire. A teaching session was also part of the intervention, but its individual impact could not be measured. Additionally, children were excluded, as the red flag features assessed, particularly contact lens use, are less relevant in paediatric populations. Including children could have skewed the documentation of these red flags, as they are not commonly applicable to this age group. This exclusion may limit the generalisability of the findings to the broader primary care population, particularly in settings where paediatric cases are more prevalent. These are acknowledged limitations and areas for improvement in future audits.

In general, larger, multi-centre audits could provide more robust data and evaluate whether improvements are sustainable over time. Additionally, further research is needed to explore patient outcomes following conservative management versus antibiotic treatment.

## Conclusions

This audit demonstrates that simple, low-cost interventions can significantly improve the assessment of red flag symptoms in patients presenting with acute red eye. However, antibiotic prescribing remains high despite limited evidence of bacterial infection. Future initiatives should prioritise sustaining improvements in clinical assessment while promoting evidence-based, conservative management to reduce unnecessary antibiotic use and mitigate the impact of drug shortages.

## Figures and Tables

**Figure 2:**
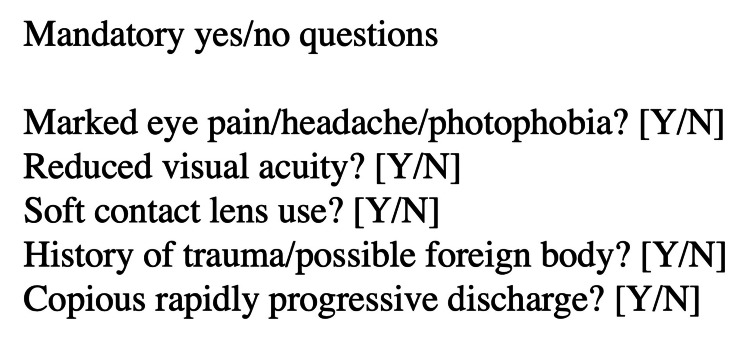
Acute red eye consultation template EMIS template for acute red eye patients under the shortcut "conjunctivitis" EMIS: Egton Medical Information Systems

**Figure 3:**
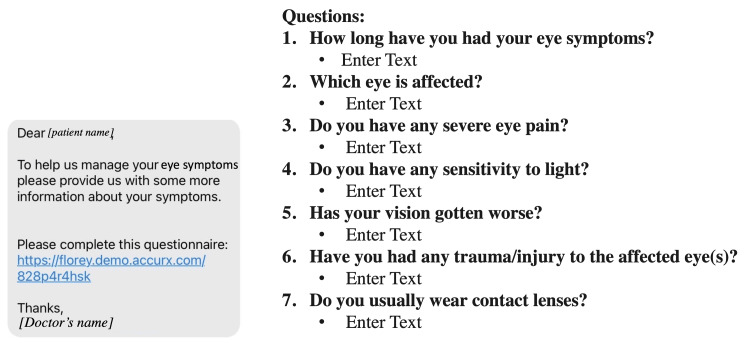
Accurx text message and questionnaire Automatic text message and questionnaire sent to patients with acute red eye presenting via telephone consultation

## Appendices

Figure 2 shows the standardised template for acute red eye consultation.

Figure 3 shows the Accurx text message and questionnaire sent to patients to fill in prior to consultation.
